# Mechanism Assessment of Physician Discourse Strategies and Patient Consultation Behaviors on Online Health Platforms: Mixed Methods Study

**DOI:** 10.2196/54516

**Published:** 2025-03-19

**Authors:** Menglei Kong, Yu Wang, Meixuan Li, Zhong Yao

**Affiliations:** 1 School of Economics and Management Beihang University Beijing China; 2 School of Public Health Capital Medical University Beijing China

**Keywords:** online health consultation, physician discourse strategies, online physician-patient trust, shared decision-making, patient consultation behavior

## Abstract

**Background:**

Online health platforms are currently experiencing significant growth. Patients can conveniently seek medical consulting services on such platforms. Against the backdrop of the thriving development of digital health care, the patterns of physician-patient communication are undergoing profound changes. It is imperative to focus on physician discourse strategies during online physician-patient interactions, which will improve the efficiency of physician-patient communication and achieve better management of the physician-patient relationship.

**Objective:**

This study aims to explore the influencing mechanism between physician discourse strategies and patient consultation behavior on online health platforms. Additionally, we explore the crucial mediating role of online physician-patient trust and the moderating role of shared decision-making in the online physician-patient communication process.

**Methods:**

We used a mixed research approach to explore the influencing mechanism. Data on physician basic attributes and physician-patient communication text records were collected from the Chunyu Doctor website using a web spider. The study obtained a total of 8628 interaction texts from January 2022 to July 2023. Physician discourse strategies (capacity-oriented strategy, quality-oriented strategy, and goodwill-oriented strategy), online physician-patient trust, and shared decision-making were captured through text mining and a random forest model. First, we employed text mining to extract the speech acts, modal resources, and special linguistic resources of each record. Then, using a well-trained random forest model, we captured the specific discourse strategy of each interaction text based on the learned features and patterns. The study generated 863 groups of physician samples with 17 data fields. The hypotheses were tested using an “ordinary least squares” model, and a stability test was conducted by replacing the dependent variable.

**Results:**

The capacity-oriented strategy, goodwill-oriented strategy, and quality-oriented strategy had significant effects on patient consultation behavior (β=.151, *P*=.007; β=.154, *P*<.001; and β=.17, *P*<.001, respectively). It should be noted that the anticipated strong effect of the capacity-oriented strategy on patient consultation behavior was not observed. Instead, the effects of the quality-oriented strategy and goodwill-oriented strategy were more prominent. Physician notification adequacy from shared decision-making moderated the effect between the goodwill-oriented strategy and patient consultation behavior (β=.172; *P*<.001). Additionally, patient expression adequacy from shared decision-making moderated the effect between the capacity-oriented strategy and patient consultation behavior (β=.124; *P*<.001), and between the goodwill-oriented strategy and patient consultation behavior (β=.104; *P*=.003). Online physician-patient trust played a significant mediating role between physician discourse strategies and patient consultation behavior.

**Conclusions:**

The study findings suggest significant implications for stimulating patient consultation behavior on online health platforms by providing guidance on effective discourse strategies for physicians, thus constructing a trustworthy physician image, improving the physician-patient relationship, and increasing platform traffic.

## Introduction

### Background

Digital health service resources in China have experienced accelerated expansion, with over 26.7 million people receiving online medical services in 2022 [1]. Notably, online health platforms, such as “Haodf” [2], “Chunyu Doctor” [3], and “JD Health” [4], in China have emerged to offer online medical services, such as health condition assessment and treatment planning. Patients primarily use online health platforms to communicate with suitable physicians about their health conditions. This behavior is commonly called “patient consultation behavior.”

Compared with offline consultation, the emergence of online health platforms has helped foster a 2-way, interactive, and democratic environment for both physicians and patients [5], but new problems have emerged. The patient consultation behavior during the online physician-patient communication process is invisible, and patients often exhibit higher levels of anxiety and eagerness to receive valuable health suggestions [6]. Moreover, it is impossible to ensure a constant online presence for both physicians and patients. These aspects may result in the quality of online consultation services for patients being compromised on the platform.

Ancient Greek scholar Hippocrates, the father of Western medicine, said, “There are two elements in the world effective of curing disease: medicine and language.” In other words, the language used by physicians plays a crucial role in enhancing the treatment effectiveness and ensuring patients’ well-being. At the beginning of online health consultation, patients select the physician who can fulfill their health care demands in terms of their disease, health condition, and preferred communication style. The physician’s title and history of peer-patient consultation are important references for potential patients to make consulting decisions. The texts entered by physicians in the chat box often encompass the diagnosis of patients’ conditions, their concerns and empathy, and the recommended treatment plans. It is crucial for physicians to communicate with patients smoothly and trustworthily by optimizing their discourse strategies, which is beneficial for stimulating patient consultation behavior. Research on physician discourse strategies in online medical consultation can provide insights into the relationship management between physicians and patients [7]. Some scholars analyzed the construction of the physician image from the perspective of language application by using qualitative research methods. It has been proven that physicians can help patients comprehend their health conditions by using reasonable, logical, and well-intended language, enabling shared decision-making and fostering rapport between both parties [8-10]. However, few studies have specifically explored the impact mechanism of physician discourse strategies on patient consultation behavior from the perspective of pragmatics, with the use of quantitative analysis.

Unlike the interaction process on other platforms, online health platforms require attentive and thorough services at every stage. Maintaining a strong and trustworthy relationship between physicians and patients greatly benefits the health management quality of patients. The “virtualized,” “fragmented,” and “casual” communication nature during online health consultation can exacerbate tensions in the physician-patient relationship [11]. These platforms and physicians face the critical stage of establishing trust. Thus, it becomes essential to consider patient consultation behavior from the perspective of trust. In addition, shared decision-making is a personalized diagnosis and communication method that was introduced by Veatch [12] in 1972. Veatch argued that shared decision-making would greatly impact the quality of physician-patient communication and medical care. Physician-patient shared decision-making is considered crucial for strengthening trust, improving communication, and enhancing the effectiveness of medical diagnosis [13]. Consequently, shared decision-making could be a significant factor affecting the relationship between discourse strategies and patients’ online consultation behavior.

Our research aims to contribute to the existing literature on patient consultation behavior by addressing 2 research objectives. First, we aim to investigate the correlation between physician discourse strategies and patient consultation behavior. Second, we seek to determine the significance of online physician-patient trust and shared decision-making in relation to the interplay between physician discourse strategies and patient consultation behavior. Based on these research objectives, we propose the following three research questions:

1. What is the relationship between physician discourse strategies and patient consultation behavior?
2. How do physician discourse strategies influence patient consultation behavior by influencing the construction of online physician-patient trust?
3. What role does shared decision-making play between physician discourse strategies and patient consultation behavior?

### Research Model and Hypotheses

#### Physician Discourse Strategies and Patient Consultation Behavior

In 1984, Frankel [14] defined physician-patient communication as the “process of exchanging information between a doctor and a patient, which is influenced by various psychological factors and their interactions.” Multiple studies have focused on the direct effects of communication style on communication outcomes [15]. We also identified that physicians often use linguistic and discursive tools to convey medical suggestions and express empathy, which can profoundly influence patients’ decision-making during consultations. We refer to these communication strategies as “physician discourse strategies.”

The SOR (stimulus, organism, and response) model was proposed by Mehrabian and Russell [16] in 1974. This psychological theory explains that environmental factors act as stimuli (S) that first influence an individual’s psychological state (O), which in turn affects their behavioral responses (R) [17,18]. In the context of online health platforms, patients encounter a wealth of external information (eg, titles, service volume, prices, etc) before and during interactions, which can stimulate their engagements, including patient consultation behavior [5,18-20]. Aligning with the SOR theory, we examined the discourse strategy employed by physicians as external stimuli received by patients, exploring their potential impact on patient consultation behavior.

Among the essential elements of these texts, language shapes not only the content of the message (ie, what is said?) but also the style (ie, how is it said?) [21]. Most studies addressed how physicians’ communication skills during consultation interactions (eg, response speed [22], interaction depth [23], and interaction rounds [24]) can shape patients’ perceptions and influence their behavioral outcomes. Unskillful responses to patients’ inquiries can not only place extra burden on physicians but also discourage patients from seeking consultation services online [21]. Additionally, scholars found that a physician with high-quality content responses [25-27], proper emotional expressions [26], the right response time [22,25], and in-depth interactions [25,28], can support a patient’s willingness to consult on online health platforms. These findings indicate that the appropriate use of discourse strategies is crucial and necessary, especially in the online interaction environment. However, these studies about physician discourse strategies are still in their infancy. Existing research has primarily focused on the superficial characteristics during physician-patient communication, neglecting the complexity of the discourse strategies employed and the foundational structures that inform them. In the study by Wu et al [27], discourse strategies were constructed according to the response time, detailed style, and emotional comfort, with the aim of investigating their effects on patient consultation satisfaction. We have incorporated insights from this study into our own research, but we delve deeper into the application of linguistic resources within physician discourse strategies. Based on the SOR theory and existing research, our research first focuses on the impact of physician discourse strategies on patient consultation behavior. We propose the following hypothesis: different types of physician discourse strategies have varying positive effects on patient consultation behavior (H1).

Trust is crucial for effective physician-patient communication, and online health platforms must prioritize building patients’ trust, which helps potential patients transition from curious observers to customers who are willing to use the platform for health consultation [29]. The trust theory proposed by Mayer et al [30] in 1995 identifies trust in 3 main dimensions: capability, quality, and goodwill. In line with the trust theory by Mayer et al [30], we categorized discourse strategies into the following: capacity-oriented strategy, quality-oriented strategy, and goodwill-oriented strategy.

The capacity-oriented strategy indicates that physicians highlight their professional skills and experiences, and show personal authority in the communication process [31]. Demonstrating such professional competencies can foster patient consultation behavior [32]. The goodwill-oriented strategy reflects that physicians are patient-centered, fulfill the needs of patients, patiently guide patients to overcome their negative emotions, and comfort their fragile hearts during communication. Empathetic communication that is easily understood by the patient can help manage expectations, ensure follow-up and treatment adherence, and improve the perception of the entire health care experience [33,34]. Such strategies may be conducive to fostering a willingness among patients to use the platform for health consultations. The quality-oriented strategy emphasizes that physicians focus on their professional code and show sincerity, honesty, and reliability in the communication process. By conveying such a communication style, physicians can foster a sense of professional quality in their interactions, thereby increasing patient trust and willingness to engage [35,36].

In combination with H1, we propose the following hypotheses: the capacity-oriented strategy enhances patient consultation behavior (H1a); the goodwill-oriented strategy enhances patient consultation behavior (H1b); and the quality-oriented strategy enhances patient consultation behavior (H1c).

#### Physician-Patient Trust

Trust is a complex dynamic that exists among individuals, organizations, and events, involving uncertainties and expectations regarding the future actions of one party by another [37,38]. Most physicians and patients communicate and interact by sending texts or audio in the chat box. Moreover, given the constraints of the online environment and the offline working hours of physicians, it is impossible to ensure a constant online presence for both physicians and patients. Patients need to share their health conditions with physicians, even those involving some private information. The establishment of trust may determine whether the patient consults and honestly shares necessary information with the doctor [39]. Thus, trust could be even more critical in the online health care environment due to individuals’ higher levels of information sensitivity and the existence of uncertainty and a lack of regulation [11].

Online trust is generated on an internet platform, where the trustor possesses attributes that benefit the trustee [40]. Online trust (eg, trust in websites, online news, and social networking site providers) has been studied extensively [41-44]. Notably, some studies have revealed that online trust strongly influences online purchase behavior [45]. In the health care sector, the influencing factors of trust are commonly studied in online health platforms, but there is less discussion on the influence of online trust on patient behavior. The establishment of trust between physicians and patients on online health platforms is often influenced by the physician’s title, gender, and reputation [46-48]. A physician’s service quality, empathy expression, average word count, and cumulative number of sessions, and the physician-patient session ratio significantly impact physician-patient trust [49,50]. Patient self-disclosure of personal information during consultations leads to increased satisfaction and trust [51,52]. The attributes, quantity, length, sentiment orientation, and composition of negative feedback in physician-patient interactions similarly influence the formation of trust between physicians and patients.

Therefore, in line with the SOR model, we conducted supplementary research on the mediating role of online physician-patient trust in the interaction between physician discourse strategies and patient consultation behavior. Hence, we propose the following hypotheses regarding the mediating role of online trust: online physician-patient trust mediates between physician discourse strategies and patient consultation behavior (H2); online physician-patient trust mediates between the capacity-oriented strategy and patient consultation behavior (H2a); online physician-patient trust mediates between the goodwill-oriented strategy and patient consultation behavior (H2b); and online physician-patient trust mediates between the quality-oriented strategy and patient consultation behavior (H2c).

#### Shared Decision-Making

The concept of shared decision-making was initially introduced by Veatch [12] in 1972 in the article “A Model for Medical Ethics in a Revolutionary Era.” Later, in 1997, Charles et al [53] further elaborated on the meaning of shared decision-making and proposed the following four crucial elements that it should encompass: (1) the participation of 2 parties; (2) the exchange of information between both parties; (3) the achievement of consensus on the treatment plan; and (4) the implementation of specific actions by both parties. Since then, shared decision-making has gained significant attention in the medical field, primarily due to the increasing emphasis on patients’ right to access information and their demand for satisfactory medical care [53]. Shared decision-making promotes the transformation of the patient’s role from a passive recipient of medical care to an active participant and supervisor, fully embracing the concept of “patient centeredness.”

Shared decision-making is a common topic in physician-patient communication and relationship management, which has been widely accepted and applied in the medical field. Some studies have proven the significance of shared decision-making in the quality and process of physician-patient communication by using qualitative methods [54,55]. Veatch [12] argued that shared decision-making would greatly impact the quality of physician-patient communication and medical care. Both patients and physicians tend to overestimate the potential benefits of interventions, and shared decision-making has been proven to be useful in influencing expectations and beliefs [56,57]. Some scholars also developed shared decision-making assessment scales [58-60] and explored the factors influencing participation in shared decision-making to gain better behavioral outcomes [61,62].

Shared decision-making could be a significant factor affecting the relationship between discourse strategies and patient online consultation behavior. However, the research issue of how shared decision-making affects the shaping process of patient behavior has not been solved, especially in the online health care environment. Under the shared decision-making philosophy, 2-way communication helps to prevent the distortion or loss of information that can result from 1-way communication, ensuring that decisions are more aligned with the best interests of the patient. Through shared decision-making, patients can gain a more comprehensive understanding of their condition and treatment options, thereby enhancing their adherence to the prescribed treatment regimen [58] and encouraging high-quality communication and patient well-being. Shared decision-making may indirectly moderate the impact of physician discourse strategies on patient consultation behavior by enhancing patient engagement, regulating physician discourse strategies, and improving medical communication.

In this study, our research objective was to clarify how shared decision-making moderates the influence of physician discourse strategies on patient consultation behavior in online health platforms, and thus, the following hypotheses are proposed: shared decision-making moderates the effect of physician discourse strategies on patient consultation behavior (H3); shared decision-making moderates the effect of the capacity-oriented strategy on patient consultation behavior (H3a); shared decision-making moderates the effect of the goodwill-oriented strategy on patient consultation behavior (H3b); and shared decision-making moderates the effect of the quality-oriented strategy on patient consultation behavior (H3c).

**Figure 1:**
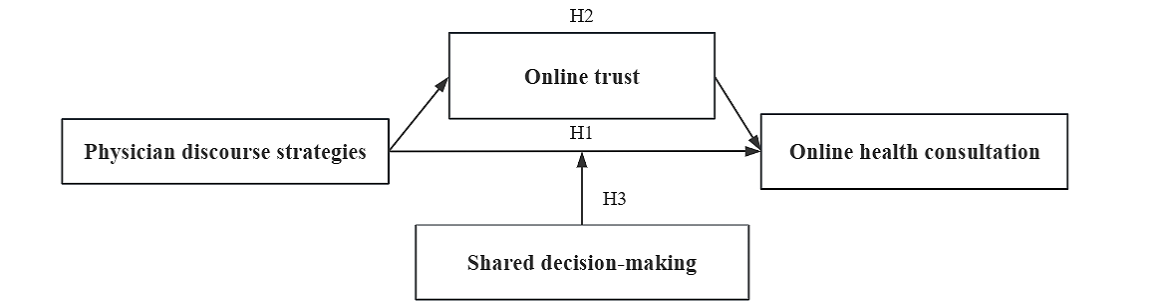
Research model. H1: different types of physician discourse strategies have varying positive effects on patient consultation behavior; H2: online physician-patient trust mediates between physician discourse strategies and patient consultation behavior; H3: shared decision-making moderates the effect of physician discourse strategies on patient consultation behavior.

Figure 1 shows our research model. The research explores the relationship between physician discourse strategies and patient consultation behavior on online health platforms (H1 and H1a-H1c), the possible mediating role of online physician-patient trust (H2 and H2a-H2c), and the moderating role of shared decision-making (H3 and H3a-H3c) in the above process.

## Methods

### Data Collection

Registered physicians on the Chunyu Doctor website are the research subjects of this paper. Chunyu Doctor, a platform with over 660,000 physicians from governmental hospitals, offers medical services to over 140,000,000 patients.

Our research explores the relationship between physician discourse strategies and patient consultation behavior while also contemplating the influences of online physician-patient trust and shared decision-making. We collected data in 2 main categories. The first category involved the basic attributes of physicians, such as gender and title. The second category involved the texts extracted from online physician-patient communication records. These anonymized patient consultation records are publicly accessible and serve as typical cases for our research [63]. We used a web spider to crawl and collect physician-patient interaction data from the “Positive Q&A” module on the Chunyu Doctor website from January 2022 to July 2023. These interaction data include information, such as consultation time, topic summary, and specific consultation text records. In total, we collected the basic attribute data of 863 physicians and 10 communication text records displayed by each doctor (8628 groups of physician-patient communication texts in total). These data cover a wide range of medical specialties, including 15 first-level departments and 43 second-level departments, with 201 groups of data per second-level department. Taking the pediatrics department as an example, we collected the attribute data of 40 physicians in the neonatology and pediatrics departments and 400 interactive text records. These texts were very complete, recording the whole physician-patient communication process.

### Ethical Considerations

This study has been reviewed and approved by the Institutional Review Board of the School of Economics and Management, Beihang University (BE-202306082). All research procedures comply with the ethical guidelines and standards set by the Institutional Review Board. The data employed in this study were deidentified interaction texts from patients, ensuring that all personal information had been anonymized in accordance with ethical guidelines. Some private data, such as the patient’s name, private disease data, and image examination records, were hidden.

### Variable Measurements

The main variables examined in this study were discourse strategies, patient consultation behavior, online physician-patient trust, and shared decision-making. The description and measurement of each variable are presented in Table 1.

**Figure 2:**
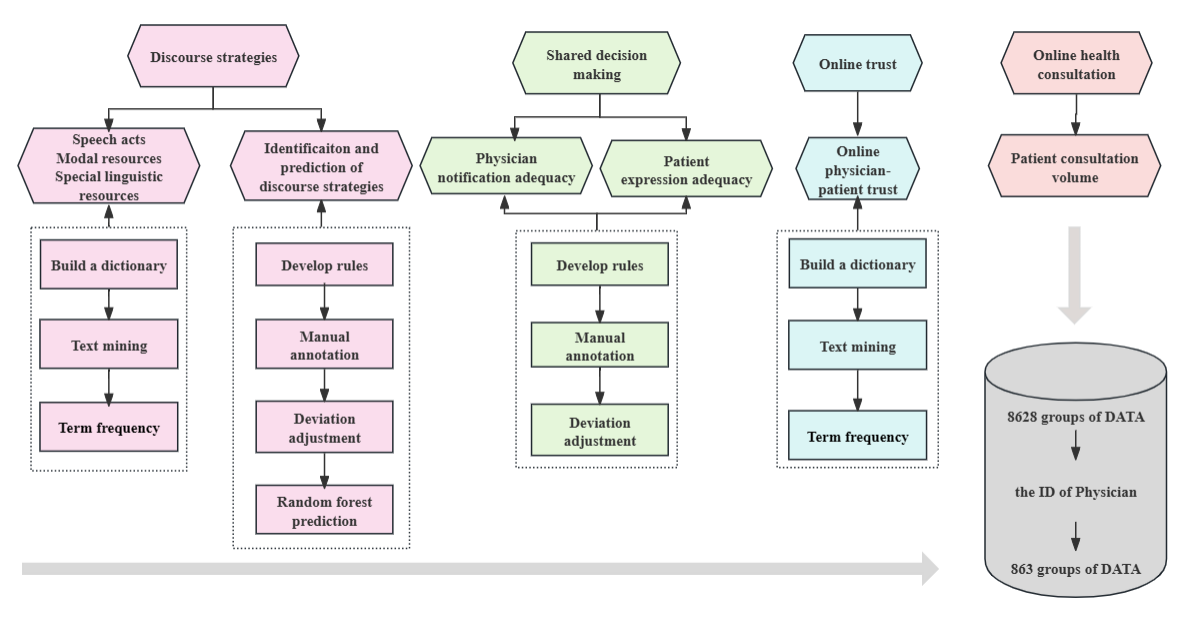
Data processing.

For the measurement of these variables, we employed text mining to analyze the physician-patient interaction texts that were scraped and applied random forest to predict the specific discourse strategy used by physicians. The data processing for all variables is illustrated in Figure 2, which will be elaborated upon in detail below. Given that the focus of this research is the physician, the data must be matched and integrated individually according to the physician ID. The integration method (summation or averaging) was employed to depict different variables regarding the performance of each physician. Ultimately, a total of 8628 data groups were integrated on a one-to-one correspondence basis with physicians, resulting in a sample dataset of 863 physicians. Each dataset included 17 data fields.

**Table 1:** Variables and their measurements.

Variables and dimensions				Measurements
**Dependent variables**				
	**Patient consultation behavior**			
		Patient consultation volume	It represents the number of physicians (*i*) offering online consultation service in (*t*) time.	
		Patient satisfaction	It represents patients rating their physicians based on their consultations.	
**Independent variables**				
	**Gender**			
		Male	1	
		Female	2	
	**Title**			
		Resident physician	1	
		Attending physician	2	
		Associate chief physician	3	
		Chief physician	4	
	**Department**			
		First-level department	It is divided into 15 first-level departments, including obstetrics, pediatrics, internal medicine, surgery, etc.	
**Analyzing variables**				
	**Discourse strategies**			
		Capacity-oriented strategy^a^	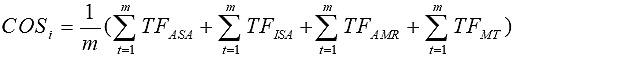 Physician (*i*) highlights professional skills and experiences, and shows personal authority in (*t*) time.	
		Goodwill-oriented strategy^b^	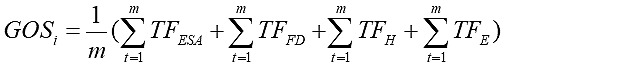 Physician (*i*) is patient-centered, fulfills the needs of patients, patiently guides patients to overcome their negative emotions, and comforts their fragile hearts in the communication process in (*t*) time.	
		Quality-oriented strategy^c^	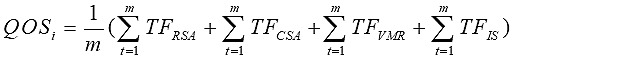 Physician (*i*) emphasizes that physicians focus on their professional code and show sincerity, honesty, and reliability in (*t*) time.	
	**Shared decision-making**			
		Physician notification adequacy	Physicians should be fully informed about alternative treatment options, including their advantages and disadvantages. Take the value of 1 if physicians exhibit the above characteristics; otherwise, take the value of 0.	
		Patient expression adequacy	Patients should openly communicate their views, concerns, values, and preferences to the physician. Take the value of 1 if patients exhibit the above characteristics; otherwise, take the value of 0.	
	**Trust**			
		Online physician-patient trust	The term frequency of words, such as “trustworthy,” “enthusiastic,” “conscientious,” and “effective,” is used to indicate the patient’s recognition of the treatment plan provided by the physician.	

^a^The abbreviations for the equation are as follows: COS: capability-oriented strategy; ASA: assertive speech acts; ISA: indicative speech acts; AMR: affirmative modal resources; MT: medical terminology.

^b^The abbreviations for the equation are as follows: GOS: goodwill-oriented strategy; ESA: expressive speech acts; FD: first-person deixis; H: honorifics; E: emojis.

^c^The abbreviations for the equation are as follows: QOS: quality-oriented strategy; RSA: refusal speech acts; CSA: committed speech acts; VMR: vague modal resources; IS: interrogative sentences.

#### Discourse Strategies

We classified discourse strategies into 3 categories: capacity-oriented strategy, quality-oriented strategy, and goodwill-oriented strategy, which were derived from the trust theory by Mayer et al [30]. To describe discourse strategies by the linguistic characteristics of speech acts, modal resources, and special linguistic resources, we have provided details in Table 2.

In describing discourse strategies, Searle and Vanderveken [64] proposed the speech act theory and categorized speech acts into 5 types based on grammatical indicators, including assertive speech acts, indicative speech acts, refusal speech acts, expressive speech acts, and committed speech acts [64,65]. The use of modal resources can also impact physician-patient communication quality [26]. First-person deixis, emojis, honorifics, and other special linguistic resources often appear in the consultation texts. The inclusion of first-person deixis facilitates the psychological convergence between physicians and patients, effectively shortening the psychological distance between the 2 parties [66]. Additionally, the appropriate use of medical terminology demonstrates the physician’s professional proficiency, fostering trust among patients [67,68]. Therefore, this paper proposes that discourse strategies used by physicians are communication strategies that appear during physician-patient interactions, regulating the way physicians exchange information and convey their emotions through the use of speech acts, modal resources, and special linguistic resources.

We calculated term frequencies corresponding to the variables according to dictionaries, which were divided into 2 categories. The first involved summarizing frequently occurring words in the physician-patient interaction texts and using the Chinese word segmentation dictionary to establish the speech act dictionary, modal resource dictionary, and special linguistic resources dictionary. The second was used to calculate the term frequency of medical terminology. We used a Chinese medical vocabulary dictionary with approximately 18,000 words, covering various dimensions, such as symptoms, drugs, medical tests, and diseases.

We then identified the discourse strategies of each text. To achieve this, we used a combination of artificial labeling and random forest for discourse strategy identification. Initially, we formulated the mathematical and semantic features of these discourse strategies. Subsequently, we analyzed the mean value of the data fields grouped by physician ID. To ensure robustness, we enlisted the help of 5 experts, either working in the medical field or studying medical-related majors. They grouped all interaction texts according to first-level departments and randomly selected 100 groups of texts for strategy identification. Each of the 5 experts independently labeled the sampled data for the 15 groups of first-level departments. To gauge the consistency of strategy identification, the identification results were cross-checked against 5 copies of each text, and the identification rules were revised accordingly. The iterative process was repeated until the labeling differences were negligible. Overall, the final average consistency for each first-level department approached over 95%.

**Table 2:** Linguistic characteristics index system.

Linguistic characteristics and index		Description
**Speech acts**		
	Assertive speech acts	Physicians judge the patient’s condition, medication, diagnosis plan, etc.
	Indicative speech acts	Physicians indicate the patient to do something.
	Refusal speech acts	Physicians express denial and disapproval of something.
	Committed speech acts	Physicians promise to do something.
	Expressive speech acts	Physicians express empathy and reassurance for the patient’s condition, experience, etc.
**Modal resources**		
	Affirmative modal resources	Physicians use words with a strongly positive attitude.
	Vague modal resources	Physicians use words with an ambiguous attitude.
**Special linguistic resources**		
	Interrogative sentences	Term frequency of the words used by physicians to express a questioning tone.
	First-person deixis	Term frequency of first-person indicative words used by physicians such as “I” and “we.”
	Honorifics	Term frequency of the words used by physicians to express respect for patients.
	Emojis	Frequency of emojis used by physicians.
	Medical terminology	Term frequency of specialized words used by physicians such as disease, medication, symptoms, and diagnosis.

Finally, a random forest algorithm was used to predict the remaining unlabeled samples. Overall, 20% of the data items with discourse strategy labels were used as test sets and 80% were used as learning sets. The algorithm had good classification performance and met the classification requirements, with a test accuracy of 85.1% and *F*-score of 0.915. The data items with blank discourse strategies were predicted and filled. Using the well-trained random forest model, we predicted the unlabeled samples in the 8628 interaction texts. The model automatically filled in the corresponding discourse strategy labels for each unlabeled interaction text based on the learned features and patterns. This process not only improved the efficiency of data annotation but also provided a more comprehensive data foundation for subsequent analysis.

Additionally, we have presented examples of physician-patient interaction texts that exemplify each of the 3 main discourse strategies to enhance readers’ comprehension of their specific meanings in Multimedia Appendix 1.

#### Online Physician-Patient Trust

We established an online trust expression word dictionary and calculated the level of online trust between physicians and patients by analyzing the frequency of relevant expression words in patient texts. Words, such as “trustworthy,” “enthusiastic,” “conscientious,” and “effective” were used to indicate the patient’s recognition of the treatment plan provided by the physician.

#### Patient Consultation Behavior

Most of the research on patient consultation often uses patient conversion rates, service volume, and patient satisfaction as feedback [25,69,70]. To measure patient consultation behavior, we used physician consultation volume on the Chunyu Doctor website, which is part of the basic physician attribute data.

#### Shared Decision-Making

The concept of shared decision-making highlights 2 important aspects. First, physicians should be fully informed about alternative treatment options, including their advantages and disadvantages. Second, patients should openly communicate their views, concerns, values, and preferences to physicians. Therefore, 2 primary indicators of shared decision-making were designed to encompass the above meanings: physician notification adequacy and patient expression adequacy.

The measurement of shared decision-making was mainly conducted through the formulation of label rule development, manual labeling, and bias adjustment. Initially, we proposed the key labeling rules of shared decision-making based on a literature search, medical knowledge, and text comprehension. Subsequently, the texts were organized and grouped within each first-level department. Five experts, either working in the medical field or studying medical-related majors, were selected to assess the 2 indicators of shared decision-making for each text in each department.

### Modeling and Statistical Analysis

#### Ordinary Least Squares Model

Before testing the main effects, moderating effects, and mediating effects, we conducted a general descriptive analysis to describe the basic attributes of physicians and the textual information on physician-patient interactions. Subsequently, a bivariate correlation model was used to test the correlations between patient consultation behavior and other variables.

We then proceeded with the test of main effects, which primarily explored the influence of discourse strategies (capacity-oriented strategy, quality-oriented strategy, and goodwill-oriented strategy) on patient consultation behavior. Additionally, we conducted the test of moderating effects, which explored the influence of shared decision-making on patient consultation behavior, and the test of mediating effects, which examined the mediating role of online physician-patient trust between discourse strategies and patient consultation behavior. The empirical models pertaining to these tests were as follows:

Main effects model: *Patient consultation behavior_i_* = *β*_0_ + *β*_1_ *COS_i_* + *β*_2_ *GOS_i_* + *β*_3_ *QOS_i_* + *β*_4_ *Control* + *ε*

Mediating effects model: *Ln Patient consultation behavior_i_* = *α*_0_ + *Ln Discourse stategies_i_*; *Ln w* = *β*_0_ + *β*_1_ *Ln Discourse stategies_i_* + *ε*; *Ln tPatient consultation behavior_i_* = *β*_0_ + *β*_1_ *Ln Discourse stategies_i_* + *β*_2_ *Ln Online physician-patient trust_i_* + *ε*

Moderating effects model: *Patient consultation behavior_i_* = *β*_0_ + *β*_1_ *COS_i_* + *β*_2_ *GOS_i_* + *β*_3_ *QOS* + *β*_4_ *COS_i_* × *SDM_i_* + *β*_5_ *GOS_i_* × *SDM_i_* + *β*_6_ *QOS_i_* × *SDM_i_* + *β*_7_ *Control* + *ε*

Control variables include the gender, title, and department of the physician. ε represents the error term. Previous research has shown that physician gender can impact patient interactions, communication styles, and potentially patient satisfaction [46,71,72]. The title of a physician can influence patient perceptions and expectations [72]. Patients may respond differently based on the physician’s level of experience or expertise, which could affect their consultation behavior. In addition, physicians from different departments may have varying approaches to communication and may encounter different patient demographics or types of consultations [73].

Next, we analyzed the data using the ordinary least squares regression model to test for main effects, moderating effects, and mediating effects. Stata (StataCorp) was used for the analysis, and all variables were standardized in the regression.

## Results

### Descriptive Statistics

Our dataset encompassed 863 physicians providing online health consultation services to 77,248 patients. The online health consultations specifically pertain to 15 first-level departments, including internal medicine, surgery, obstetrics, pediatrics, oncology, and prevention and treatment. Among the 863 physicians, there were 637 male physicians and 226 female physicians from 15 departments. The plastic and cosmetic department had the fewest physicians (n=18), while the surgery department had the most physicians (n=197). Attending physicians had the highest count (n=411), while chief physicians had the lowest count (n=83). The 863 physicians had an average of 7847.81 total patient consultations and a mean patient satisfaction rate of 98.53%. Among the 3 types of discourse strategies, the largest number of physicians (n=347) employed the capacity-oriented strategy. Moreover, 411 physicians on the online health platform were primary care physicians. The descriptive statistics are shown in Table 3.

**Table 3:** Descriptive statistics.

Variable			Value (N=863)
Patient consultation volume, mean (SD)			7847.81 (12,252.30)
Online physician-patient trust, mean (SD)			4.92 (4.30)
**Shared decision-making, mean (SD)**			
	Physician notification adequacy	3.10 (2.72)	
	Patient expression adequacy	6.70 (3.71)	
**Physician discourse strategies, mean (SD)**			
	Capacity-oriented strategy	42.06 (23.94)	
	Quality-oriented strategy	8.52 (4.92)	
	Goodwill-oriented strategy	6.28 (4.92)	
**Gender, n (%)**			
	Male	637 (73.8)	
	Female	226 (26.2)	
**Title, n (%)**			
	Resident physician	136 (15.8)	
	Attending physician	411 (47.6)	
	Associate chief physician	233 (27.0)	
	Chief physician	83 (9.6)	
**Department, n (%)**			
	Obstetrics department	20 (2.3)	
	Pediatrics department	40 (4.6)	
	Otolaryngology department	60 (7.0)	
	Gynecology department	20 (2.3)	
	Orthopedics department	60 (7.0)	
	Oral and maxillofacial department	20 (2.3)	
	Andrology department	20 (2.3)	
	Internal medicine department	180 (20.9)	
	Dermatology and sexually transmitted disease department	40 (4.6)	
	Surgery department	197 (22.8)	
	Ophthalmology department	20 (2.3)	
	Nutrition department	20 (2.3)	
	Plastic and cosmetic department	18 (2.1)	
	Traditional Chinese medicine department	89 (10.3)	
	Oncology department	59 (6.8)	

### Pearson Correlation Analysis and Collinearity Testing

As shown in Table 4, patient consultation volume had the highest positive correlation with the goodwill-oriented strategy (*r*=0.261; *P*=.008). Patient consultation satisfaction showed positive correlations with the capacity-oriented strategy (*r*=0.282; *P*=.008), quality-oriented strategy (*r*=0.271; *P*=.003), goodwill-oriented strategy (*r*=0.122; *P*=.005), and physician notification adequacy (*r*=0.187; *P*=.003).

When there is a high correlation between 2 independent variables, the existence of one variable may cause the regression coefficient of the other variable to rise sharply. Collinearity becomes a problem in regression analysis, but this does not mean that this variable has no predictive effect on the dependent variable. Because the correlation coefficient between the quality-oriented strategy and capacity-oriented strategy was high (more than 0.7), there might have been a multiple collinearity issue. Thus, this study used variance inflation factor (VIF) testing to examine the collinearity between the 2 variables.

**Table 4:** Correlations between variables.

Variables		COS^a^	QOS^b^	GOS^c^	PNA^d^	PEA^e^	OPT^f^	PCV^g^	PCS^h^
**COS**									
	*r*	1	0.765^i^	0.400^i^	0.358	0.065	0.536^i^	0.220^i^	0.282^i^
	*P* value	—^j^	.009	.005	.003	.56	.005	.003	.008
**QOS**									
	*r*	0.765^i^	1	0.391^i^	0.310^i^	0.032	0.509^i^	0.132^i^	0.271^i^
	*P* value	.009	—	.003	.005	.66	.003	.009	.003
**GOS**									
	*r*	0.400^i^	0.391^i^	1	0.237^i^	–0.005	0.488^i^	0.261^i^	0.122^i^
	*P* value	.005	.003	—	.005	.58	.003	.008	.005
**PNA**									
	*r*	0.358	0.310^i^	0.237^i^	1	0.423^i^	0.243^i^	0.243^i^	0.187^i^
	*P* value	.003	.005	.005	—	.005	.005	.008	.003
**PEA**									
	*r*	0.065	0.032	–0.005	0.423^i^	1	0.036	0.087^k^	0.126^i^
	*P* value	.56	.66	.58	.005	—	.08	.02	.004
**OPT**									
	*r*	0.536^i^	0.509^i^	0.488^i^	0.243^i^	0.036	1	0.250^i^	0.182^i^
	*P* value	.005	.003	.003	.005	.08	—	.003	.006
**PCV**									
	*r*	0.220^i^	0.132^i^	0.261^i^	0.243^i^	0.087^k^	0.250^i^	1	0.036
	*P* value	.003	.009	.008	.008	.02	.003	—	.18
**PCS**									
	*r*	0.282^i^	0.271^i^	0.122^i^	0.187^i^	0.126^i^	0.182^i^	0.036	1
	*P* value	.008	.003	.005	.003	.004	.006	.18	—

^a^COS: capacity-oriented strategy.

^b^QOS: quality-oriented strategy.

^c^GOS: goodwill-oriented strategy.

^d^PNA: physician notification adequacy.

^e^PEA: patient expression adequacy.

^f^OPT: online physician-patient trust.

^g^PCV: patient consultation volume.

^h^PCS: patient consultation satisfaction.

^i^The correlation is significant at a significance level of .001 (2-tailed).

^j^Not applicable.

^k^The correlation is significant at a significance level of .01 (2-tailed).

In the regression model, the VIF can measure the severity of multicollinearity. It is generally believed that if the VIF value is greater than 10, there is a multicollinearity problem (strictly greater than 5) [74]. We conducted collinearity analysis on physician discourse strategies, the capacity-oriented strategy, the quality-oriented strategy, the goodwill-oriented strategy, physician notification adequacy, patient expression adequacy, online physician-patient trust, and patient consultation satisfaction, and found that the VIF values of these variables were 1.398, 4.768, 4.803, 1.667, 1.245, 1.510, 1.096, and 2.083, respectively. All VIF values of the variables were lower than 5, indicating that there was no serious collinearity issue among the independent variables in this study.

### Hypothesis Testing

Our research focused on the Chunyu Doctor platform, a leading online health market in China with a significant number of physician and patient registrations. We investigated the impact of physician discourse strategies on patient consultation behavior in this online health platform. To validate our research models, we first examined the relationship between physician discourse strategies (capacity-oriented strategy, quality-oriented strategy, and goodwill-oriented strategy) and patient consultation behavior. Then, we introduced moderating variables, namely physician notification adequacy and patient expression adequacy, into the model. This allowed us to test our main effects hypotheses (H1, H1a-H1c) as well as the moderating effect of shared decision-making between physician discourse strategies and patient consultation behavior (H3, H3a-H3c). The regression results are summarized in Table 5.

The capacity-oriented strategy, goodwill-oriented strategy, and quality-oriented strategy had significant effects on patient consultation behavior (β=.151, *P*=.003; β=.154, *P*<.001; and β=.170, *P*<.001, respectively). It should be noted that the anticipated strong effect of the capacity-oriented strategy on patient consultation behavior was not observed. Instead, the effects of the quality-oriented strategy and goodwill-oriented strategy were more prominent.

We then conducted a test on the moderating effects. The interaction term between the capability-oriented strategy and physician notification adequacy did not show significance (β=.062; *P*=.62), while the interaction term between the capability-oriented strategy and patient expression adequacy showed significance (β=.124; *P*<.001). This suggests that there is a significant difference in the effect of the capability-oriented strategy on patient consultation behavior at different levels of the moderating variable (patient expression adequacy).

Similarly, the interaction term between the goodwill-oriented strategy and physician notification adequacy was significant (β=.172; *P*<.001), indicating that the effect of the goodwill-oriented strategy on patient consultation behavior varies significantly at different levels of the moderating variable (physician notification adequacy).

In addition, the interaction term between the goodwill-oriented strategy and patient expression adequacy showed significance (β=.104; *P*=.003), suggesting that the effect of the goodwill-oriented strategy on patient consultation behavior differs significantly at different levels of the moderating variable (patient expression adequacy).

The interaction term between the quality-oriented strategy and physician notification adequacy did not show significance (β=.013; *P*=.23). Similarly, the interaction term between the quality-oriented strategy and patient expression adequacy did not show significance (β=.033; *P*=.42). This implies that the effect of the quality-oriented strategy on patient consultation remains consistent at different levels of the moderating variable.

**Table 5:** Impact of shared decision-making on patient consultation behavior.

Variable	Model 1 (β)	Model 2 (β)	Model 3 (β)	Model 4 (β)	Model 5 (β)
Gender	–0.003	–0.010	–0.003	0.006	–0.009
Title	0.154^a^	0.149^a^	0.143^a^	0.168	0.140^a^
Department	–0.074^b^	–0.050^b^	–0.076^b^	–0.109^a^	–0.066^b^
Capacity-oriented strategy	0.151^a^	0.159^b^	—^c^	—	0.282^a^
Goodwill-oriented strategy	0.154^d^	—	0.160^d^		0.373^a^
Quality-oriented strategy	0.170^d^	—		0.127^a^	0.222^b^
Capacity-oriented strategy × physician notification adequacy	—	0.062	—	—	0.155
Capacity-oriented strategy × patient expression adequacy	—	0.124^d^	—	—	0.780^a^
Goodwill-oriented strategy × physician notification adequacy	—	—	0.172^d^	—	0.704^a^
Goodwill-oriented strategy × patient expression adequacy	—	—	0.104^a^	—	0.065^a^
Quality-oriented strategy × physician notification adequacy	—	—	—	0.013	0.021
Quality-oriented strategy × patient expression adequacy	—	—	—	0.033	0.070
Constant	99.041^d^	99.148^a^	98.958^d^	98.813^d^	91.863^a^
*F*	18.204^d^	17.214^d^	24.593^d^	10.750^d^	16.907^d^
*R* ^2^	0.152	0.108	0.153	0.064	0.193

^a^The correlation is significant at a significance level of .01 (2-tailed).

^b^The correlation is significant at a significance level of .05 (2-tailed).

^c^Not applicable.

^d^The correlation is significant at a significance level of .001 (2-tailed).

**Figure 3:**
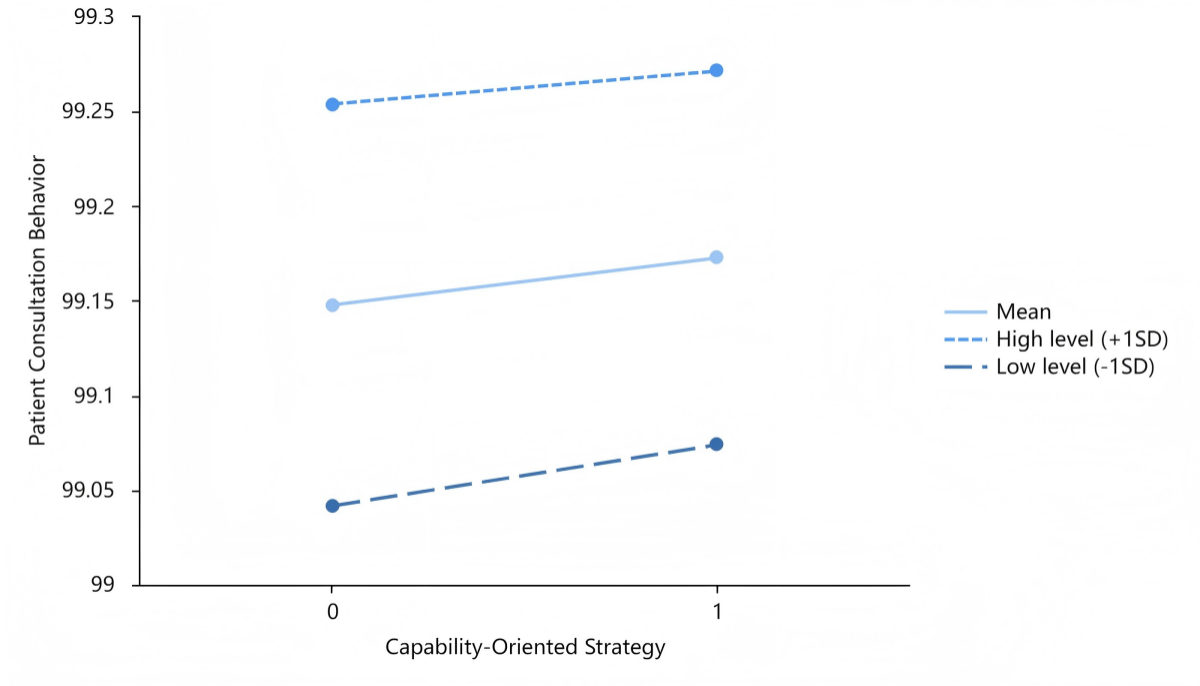
Moderating role of physician notification adequacy with the capability-oriented strategy.

**Figure 4:**
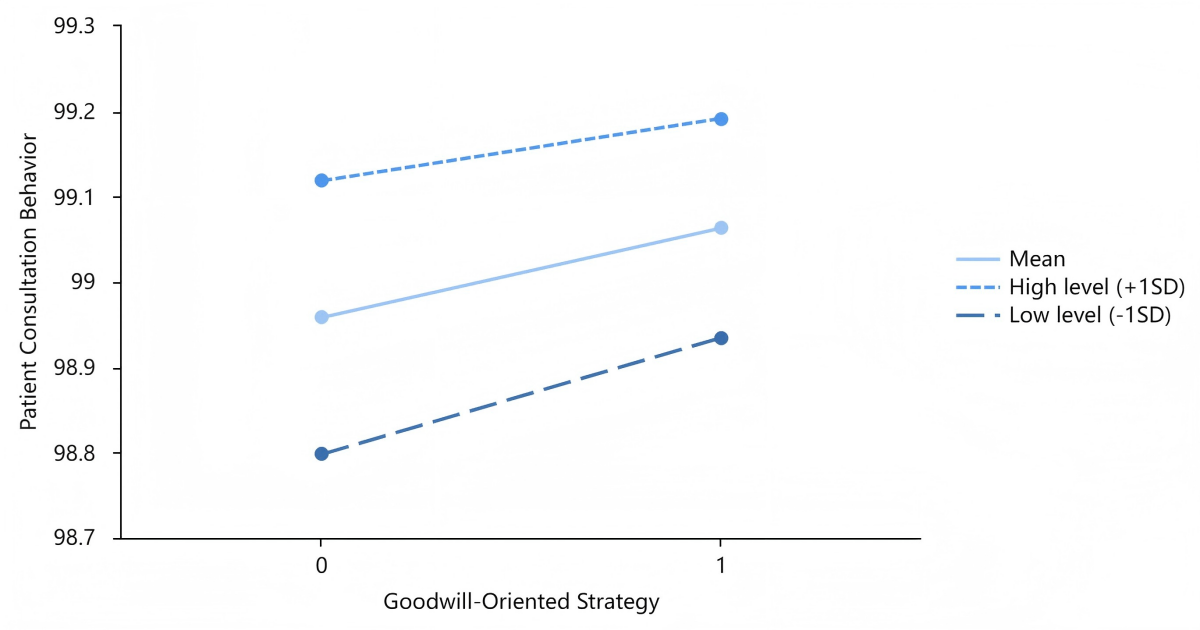
Moderating role of physician notification adequacy with the goodwill-oriented strategy.

**Figure 5:**
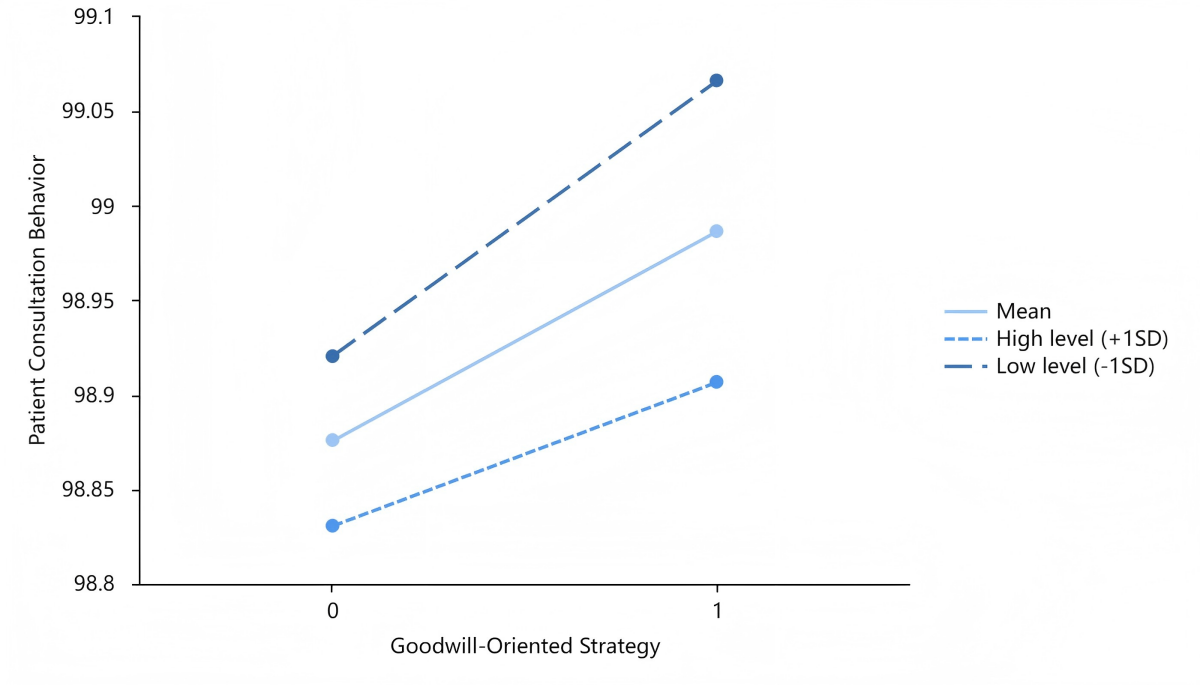
Moderating role of patient expression adequacy with the goodwill-oriented strategy.

Figure 3 depicts the moderating effects of patient expression adequacy on the interplay of the capability-oriented strategy and patient consultation behavior. Figures 4 and 5 describe the moderating roles of physician notification adequacy with the goodwill-oriented strategy and patient expression adequacy with the goodwill-oriented strategy, respectively. The moderating variables were categorized into 3 levels: mean level, high level (mean + SD), and low level (mean – SD).

Next, our study examined the mediating role of online physician-patient trust using the nonparametric percentile bootstrap method to test the mediating effects, as shown in Table 6. Physician discourse strategies significantly affected patient consultation behavior (β=.292; *P*<.001). The capacity-oriented strategy significantly affected patient consultation behavior (β=0.260; *P*<.001). The quality-oriented strategy significantly affected patient consultation behavior (β=.223; *P*<.001). The goodwill-oriented strategy significantly affected patient consultation behavior (β=.075; *P*<.001). It involved repeating the sampling 2000 times and estimating 95% CIs. If the CI does not include zero, it indicates a significant mediating effect [75]. In our analysis, online physician-patient trust (95% CI 0.724-0.743) was found to mediate the relationship between physician discourse strategies and patient consultation behavior, with a mediation effect value of 0.348. The mediating effect of online physician-patient trust on the relationship between the capacity-oriented strategy and patient consultation behavior showed a value of 0.031. Additionally, the mediating effect of online physician-patient trust on the relationship between the quality-oriented strategy and patient consultation behavior showed a value of 0.042. Moreover, the mediating effect of online physician-patient trust on the relationship between the goodwill-oriented strategy and patient consultation behavior showed a value of 0.095. Likewise, the goodness-of-fit of the mediating effect model between online physician-patient trust in the 3 dimensions of physician discourse strategies and patient consultation behavior reached the standard (*χ*^2^/df <5; comparative fit index [CFI] >0.9; Tucker-Lewis index [TLI] >0.9; root mean square error of approximation [RMSEA] <0.08; standardized root mean square residual [SRMR] <0.5).

**Table 6:** Standardized effects of model paths.

Model paths		Estimates	Boot SE	Boot LLCI^a^	Boot ULCI^b^
**Direct effect**					
	Physician discourse strategies→patient consultation behavior	0.292	0.033	0.181	0.214
	Capacity-oriented strategy→patient consultation behavior	0.260	0.028	0.017	0.031
	Quality-oriented strategy→patient consultation behavior	0.223	0.029	0.165	0.280
	Goodwill-oriented strategy→patient consultation behavior	0.075	0.028	0.017	0.129
**Mediation effect**					
	Physician discourse strategies→online physician-patient trust→patient consultation behavior	0.348	0.008	0.724	0.743
	Capacity-oriented strategy→online physician-patient trust→patient consultation behavior	0.031	0.205	0.002	0.314
	Quality-oriented strategy→online physician-patient trust→patient consultation behavior	0.042	0.018	0.004	0.076
	Goodwill-oriented strategy→online physician-patient trust→patient consultation behavior	0.095	0.017	0.061	0.129
**Total effect**					
	Physician discourse strategies→patient consultation behavior	0.640	0.034	0.737	0.753
	Capacity-oriented strategy→patient consultation behavior	0.291	0.247	0.021	0.337
	Quality-oriented strategy→patient consultation behavior	0.265	0.024	0.219	0.312
	Goodwill-oriented strategy→patient consultation behavior	0.170	0.023	0.123	0.215

^a^LLCI: lower limit confidence interval.

^b^ULCI: upper limit confidence interval.

### Robustness Testing

This section primarily aims to validate the stability of the derived model and establish the validity of the research conclusions. In this study, patient consultation volume was considered as a variable representing patient consultation behavior. However, patients on online health platforms can rate the physicians they interact with. Thus, patient consultation satisfaction is considered as an alternative variable for patient consultation behavior [25,69,70], which replaced patient consultation volume and was included in the regression analysis model. The robustness testing results are presented in Table 7, reconfirming the earlier findings and indicating the relatively stable nature of this study’s results.

The capacity-oriented strategy, goodwill-oriented strategy, and quality-oriented strategy had significant effects on patient consultation behavior (β=.169, *P*=.002; β=.274, *P*<.001; and β=.357, *P*<.001, respectively). Physician notification adequacy from shared decision-making moderated the effect between the goodwill-oriented strategy and patient consultation behavior (β=.153; *P*<.001). Patient expression adequacy from shared decision-making moderated the effect between the capacity-oriented strategy and patient consultation behavior (β=.121; *P*<.001) and between the goodwill-oriented strategy and patient consultation behavior (β=.106; *P*=.03).

Furthermore, we employed the nonparametric percentile bootstrap method to test the significance of the mediating effects. The results are shown in Table 8. This involved repeating the sampling 2000 times and estimating 95% CIs. If the CI does not include zero, it indicates a significant mediating effect. In our analysis, online physician-patient trust (95% CI 0.002-0.061) was found to mediate the relationship between physician discourse strategies and patient consultation behavior, with a mediation effect value of 0.014. The mediating effect of online physician-patient trust on the relationship between the capacity-oriented strategy and patient consultation behavior showed a value of 0.034. Additionally, the mediating effect of online physician-patient trust on the relationship between the quality-oriented strategy and patient consultation behavior showed a value of 0.038. Moreover, the mediating effect of online physician-patient trust on the relationship between the goodwill-oriented strategy and patient consultation behavior showed a value of 0.077. Likewise, the goodness-of-fit of the mediating effect model between online physician-patient trust in the 3 dimensions of physician discourse strategies and patient consultation behavior reached the standard (*χ*^2^/df <5; CFI >0.9; TLI >0.9; RMSEA <0.08; SRMR <0.5).

**Table 7:** Impact of shared decision-making on online health consultation (patient consultation satisfaction).

Variable	Model 1 (β)	Model 2 (β)	Model 3 (β)	Model 4 (β)	Model 5 (β)
Gender	–0.003	–0.078	–0.072	0.006	–0.036
Title	0.154^a^	0.030	0.045	0.168	0.160^a^
Department	–0.074^b^	–0.112	–0.111	–0.109^a^	0.011
Capacity-oriented strategy	0.169^a^	0.193^c^	—^d^	—	0.208^a^
Goodwill-oriented strategy	0.274^c^	—	0.270	—	0.185^a^
Quality-oriented strategy	0.357^c^	—	—	0.227^a^	0.117^a^
Capacity-oriented strategy × physician notification adequacy	—	0.079	—	—	0.062
Capacity-oriented strategy × patient expression adequacy	—	0.121^c^	—	—	0.106^a^
Goodwill-oriented strategy × physician notification adequacy	—	—	0.153^c^	—	0.193^a^
Goodwill-oriented strategy × patient expression adequacy	—	—	0.106^b^	—	0.097^b^
Quality-oriented strategy × physician notification adequacy	—	—	—	0.013	0.018
Quality-oriented strategy × patient expression adequacy	—	—	—	0.133	0.037
Constant	195.120^c^	286.242^a^	284.934^c^	275.443^a^	263.321^c^
*F*	15.03^c^	19.450^c^	18.225^c^	7.838^c^	8.844^c^
*R^2^*	0.152	0.114	0.153	0.045	0.119

^a^The correlation is significant at a significance level of .01 (2-tailed).

^b^The correlation is significant at a significance level of .05 (2-tailed).

^c^The correlation is significant at a significance level of .001 (2-tailed).

^d^Not applicable.

**Table 8:** Standardized effects of model paths (patient consultation satisfaction).

Model paths			Estimates		Boot SE		Boot LLCI^a^		Boot ULCI^b^
**Direct effect**									
	Physician discourse strategies→patient consultation satisfaction	0.009		0.019		0.068		0.130	
	Capacity-oriented strategy→patient consultation satisfaction	0.186		0.027		0.131		0.236	
	Quality-oriented strategy→patient consultation satisfaction	0.171		0.026		0.119		0.219	
	Goodwill-oriented strategy→patient consultation satisfaction	0.059		0.023		0.015		0.106	
**Mediation effect**									
	Physician discourse strategies→online physician-patient trust→patient consultation satisfaction	0.014		0.012		0.002		0.061	
	Capacity-oriented strategy→online physician-patient trust→patient consultation satisfaction	0.034		0.015		0.002		0.062	
	Quality-oriented strategy→online physician-patient trust→patient consultation satisfaction	0.038		0.016		0.003		0.067	
	Goodwill-oriented strategy→online physician-patient trust→patient consultation satisfaction	0.077		0.014		0.049		0.104	
**Total effect**									
	Physician discourse strategies→patient consultation satisfaction	0.113		0.017		0.086		0.140	
	Capacity-oriented strategy→patient consultation satisfaction	0.220		0.024		0.169		0.261	
	Quality-oriented strategy→patient consultation satisfaction	0.209		0.022		0.164		0.252	
	Goodwill-oriented strategy→patient consultation satisfaction	0.136		0.021		0.091		0.176	

^a^LLCI: lower limit confidence interval.

^b^ULCI: upper limit confidence interval.

## Discussion

### Principal Findings

The study confirmed that the 3 primary discourse strategies (capacity-oriented strategy, quality-oriented strategy, and goodwill-oriented strategy) used by physicians have positive impacts on patient consultation behavior. It should be noted that the anticipated strong effect of the capacity-oriented strategy on patient consultation behavior was not observed. Instead, the effects of the quality-oriented strategy and goodwill-oriented strategy were more prominent. Physician notification adequacy from shared decision-making moderated the effect between the goodwill-oriented strategy and patient consultation behavior. Patient expression adequacy from shared decision-making moderated the effect between the capacity-oriented strategy and patient consultation behavior, and between the goodwill-oriented strategy and patient consultation behavior. Online physician-patient trust played significant mediating roles between physician discourse strategies and patient consultation behavior.

### Theoretical Implications

This study addresses the gap in research on the influence of physician discourse strategies on patient consultation behavior, providing a new perspective and theoretical basis for the study of the antecedents of patient consultation behavior, especially in the online health environment. Previous studies on patient consultation behavior have mainly focused on influencing factors, such as text amount, response time, negative emotions, empathy, and information disclosure exhibited in physician texts [25,26,69,74]. Few studies have focused on the influence of discourse strategies on patient consultation behavior. Furthermore, this research extends the SOR theory, speech act theory, and trust theory by Mayer et al [30] to the study of patient behavior on online health platforms. Linguistic characteristics of physician texts are used to depict discourse strategies, including speech act features, modal resource features, and special linguistic resource features.

Moreover, the dimensions of research on the characterization of physician discourse strategies have been expanded. The study identified the significant value of managing physician discourse strategies in stimulating patient consultation behavior. Our findings demonstrate that physicians on the platform are required to have comprehensive expressions, in-depth knowledge of the patient’s health condition, and accountability to the patient. This is more important than simply showing professional competence to enhance both the quantity and quality of consulting services. The results are counterintuitive, revealing that a quality-oriented strategy has a greater positive impact on patient consultation behavior compared to a goodwill-oriented strategy and capacity-oriented strategy, which have weaker effects. The findings suggest that physician discourse strategies that minimize the use of strong words, focus on comprehensive questioning about the patient’s condition, and take responsibility for the patient’s well-being are crucial for stimulating potential patients to seek the first consultation and reconsultations. Additionally, the use of emojis, first-person deixis, and honorifics can help physicians build rapport with patients and encourage them to seek consultations. On the other hand, patients do not prefer physicians who solely demonstrate professional competence, create a greater power distance between themselves and their patients, and adopt an assertive approach in their interactions.

Building on the generalized model of online trust proposed by Shankar et al [40] in 2002 and the SOR model [17,18], this study investigated the mediating role of online physician-patient trust between physician discourse strategies and patient consultation behavior. The concept of “online trust” in e-commerce platforms has been extended to online health platforms [44]. Our findings indicate that online physician-patient trust has a mediating effect on the relationship between physician discourse strategies and patient consultation behavior, offering guidance for the platform to strategically position its future competitiveness, particularly in the construction of the physician’s image.

We divided shared decision-making into 2 dimensions based on its fundamental concept: physician notification adequacy and patient expression adequacy. Importantly, our study is the first to incorporate physician-patient shared decision-making as a moderating variable in the research of patient consultation behavior. Shared decision-making, which has gained attention in the medical field in recent years, lacks quantitative empirical research in medical practice [61,62]. Our findings revealed that physician notification adequacy only moderated the effect of the goodwill-oriented strategy on patient consultation behavior. It did not play a moderating role in the relationship between the capacity-oriented strategy and patient consultation behavior, as well as the quality-oriented strategy and patient consultation behavior. Patient expression adequacy had moderate effects on the capacity-oriented strategy and goodwill-oriented strategy in relation to patient consultation behavior, but it failed to moderate the relationship between the quality-oriented strategy and patient consultation behavior. It suggests that, when using the capacity-oriented strategy, allowing patients to express their doubts regarding their condition and treatment plan strongly impacts patient consultation behavior. This finding highlights the importance of communication barriers between physicians and patients. Under the capacity-oriented strategy, physicians pay little attention to whether patients can comprehend medical principles or professional considerations and instead adhere to the attitude of “just listen to me.” Consequently, the participation and experiences of patients are compromised. For physician-patient communication to be smooth, patients must express their doubts and accurately understand the information conveyed by the physician. Under the goodwill-oriented strategy, there are fewer communication barriers between physicians and patients, creating more opportunities for the shared decision-making dimension to have a positive impact on patient consultation behavior. Conversely, under the quality-oriented strategy, the physician’s expression has already provided a greater space for information exchange between the 2 parties. For example, using words, such as “better” and “suggest,” and employing interrogative sentences can induce patients to provide a detailed description of their health condition. As a result, the role of shared decision-making may be partially played by the quality-oriented strategy, and thus, it does not have a notable impact on patient consultation behavior.

### Managerial Implications

In terms of contributions to platform management, our results offer guidance for the platform to strategically position its future competitiveness, particularly in terms of physician image construction. This includes the use of physician discourse strategies and shared decision-making, both of which can help increase patient consultation volume. It should be noted that the term “patients” refers to not only current patients but also potential patients, and the term “physicians” also includes artificial intelligence (AI) medical chatbots [76]. If physicians respond to patients in a more appealing and effective way, the online health consultation experiences of patients will be more enjoyable and comfortable. To meet patients’ preferences for physician discourse strategies, communication training for physicians or future training of AI medical chatbots can be used to assist in physician-patient interactions, with the corpus in large language models designed accordingly. In addition, the platform should enhance the supervision and evaluation of the quality of physician-patient communication, develop scientifically formulated standards and norms for physicians to respond to patients, and improve physicians’ communication skills and abilities [28,32].

### Limitations

Regarding the classification of physician discourse strategies, it is important to note that different theories may lead to different categorizations. In this study, our focus was on how physician discourse strategies enhance patient consultation behavior by strengthening online trust between physicians and patients. The categorization of our discourse strategies is rooted in the trust theory. If future research is based on other theoretical frameworks, the understanding of physician discourse strategies will naturally be different.

To identify physician discourse strategies, our study adopted a combination of manual annotation and the random forest algorithm. This means that the “capacity-oriented strategy,” “quality-oriented strategy,” and “goodwill-oriented strategy” are determined by human judgments based on identification rules. While we designed a bias adjustment mechanism and calculated the level of bias among different judges, it is important to acknowledge that current prevalent large language models, such as the Bidirectional Encoder Representations from Transformers (BERT) algorithm and Generative Pre-trained Transformers (GPT) algorithm, may have a better understanding of semantics and higher efficiency than the manual annotation approach.

In this study, due to regulations regarding patient personal privacy on online health platforms, there were limitations in collecting data and samples. Moreover, there may have been endogenous issues like missing variables and 2-way causality, although we made some effort to solve these issues. In the future, we will conduct thorough research on these issues.

## Appendix

Multimedia Appendix 1 Characteristics of speech acts, modal resources, and special linguistic resources according to physician discourse strategies.

## Abbreviations

AI: artificial intelligence
CFI: comparative fit index
RMSEA: root mean square error of approximation
SOR: stimulus, organism, and response
SRMR: standardized root mean square residual
TLI: Tucker-Lewis index
VIF: variance inflation factor
