# Supplementary material for: Mechanism Assessment of Physician Discourse Strategies and Patient Consultation Behaviors on Online Health Platforms: Mixed Methods Study
Source: J Med Internet Res. 2025 Mar 19;27:e54516. doi: 10.2196/54516 (PMC11966082; doi:10.2196/54516)
Supplement: Multimedia Appendix 1 [file jmir_v27i1e54516_app1.docx]

**Multimedia Appendix 1.** Characteristics of speech acts, modal resources, and special linguistic resources according to physician discourse strategies.

| Discourse Strategies | Definitions and Performances | Physician-patient Interaction Examples |
| --- | --- | --- |
| Capacity-  oriented Strategy | ***COS indicates that physicians highlight their professional skills and experiences, and show personal authority in the communication process.***  In terms of speech acts, assertive and indicative speech acts were observed to be more frequently used.  Regarding modal resources, affirmative modal resources like “must” and “have to” were found to be used more frequently.  As for medical terminology, it was noticed that medical terms were used more frequently. | P1: My temperature is 37.6 degrees Celsius.  D1: You must use Chai Gui fever granules or black bean curd granules. Typically, antipyretic medications like ibuprofen and acetaminophen can be used when the temperature exceeds 38.2 degrees Celsius. |
| Goodwill-  oriented Strategy | ***GOS reflected that physicians are patient-centered, fulfill the needs of patients, patiently guide patients to overcome their negative emotions and comfort their fragile hearts in the communication process.***  In terms of speech acts, emotional words are utilized by physicians to provide comfort to patients, resulting in a higher frequency of expression speech acts.  In terms of special linguistic resources, physicians employ first-person deixis, emojis, and honorifics to express empathy and provide comfort to patients, as exemplified by phrases like "Don't be nervous, don't think too much," "Get well soon! " and so on. | D2: Hello, thanks for your trust and support!  P2: Currently, I am taking Eugenol 1+1/4 tablets and experiencing some weight gain and my breast nodules classified as category 3.  D2: You are on the right dosage if you do not plan to get pregnant. I noticed that your height and weight indicate that you are overweight. I suggest increasing protein intake and drinking warm boiled water daily. |
| Quality-  oriented Strategy | ***QOS emphasizes that physicians focus on their professional code and show sincerity, honesty and reliability in the communication process.***  In the analysis of speech acts, there is a high occurrence of refusive and committed speech acts, indicating that the physician actively engages in inquiring about the patient's condition and regular follow-up visits.  Concerning modal resources, it was found that ambiguous modal resources such as "it is better" and "it is safer" were used more frequently.  Regarding the employment of special linguistic resources, the physician primarily employs condition-seeking interrogative sentences to convey their focus. | D3: How long has this symptom been present?  P3: It started just one day ago.  D3:It will become better by using some Anti-inflammatory drugs such as cefaclor. Do you have any allergic reactions?  P3: I had an allergic reaction to cephalosporin.  D3: In that case, you should avoid cephalosporin. You will get better soon. And please return visit in time. |
| D and P represent the physician’s part and the patient’s part in physician-patient communication texts, respectively. | | |
